# Localized fine-tuning and clinical evaluation of deep-learning based auto-segmentation (DLAS) model for clinical target volume (CTV) and organs-at-risk (OAR) in rectal cancer radiotherapy

**DOI:** 10.1186/s13014-024-02463-0

**Published:** 2024-07-02

**Authors:** Jianhao Geng, Xin Sui, Rongxu Du, Jialin Feng, Ruoxi Wang, Meijiao Wang, Kaining Yao, Qi Chen, Lu Bai, Shaobin Wang, Yongheng Li, Hao Wu, Xiangmin Hu, Yi Du

**Affiliations:** 1 Department of Radiation Oncology, Key Laboratory of Carcinogenesis and Translational Research (Ministry of Education/Beijing), Peking University Cancer Hospital & Institute, Beijing, 100142 China; 2 Research and Development Department, MedMind Technology Co., Ltd, Beijing, 100083 China; 3 Institute of Medical Technology, Peking University Health Science Center, Beijing, 100191 China; 4 Beijing Key Lab of Nanophotonics and Ultrafine Optoelectronic Systems, School of Optics and Photonics, Beijing Institute of Technology, Beijing, 100081 China

**Keywords:** Deep learning, Auto-contouring, Rectal cancer, Clinical target volume, Organ at risk

## Abstract

**Background and purpose:**

Various deep learning auto-segmentation (DLAS) models have been proposed, some of which have been commercialized. However, the issue of performance degradation is notable when pretrained models are deployed in the clinic. This study aims to enhance precision of a popular commercial DLAS product in rectal cancer radiotherapy by localized fine-tuning, addressing challenges in practicality and generalizability in real-world clinical settings.

**Materials and methods:**

A total of 120 Stage II/III mid-low rectal cancer patients were retrospectively enrolled and divided into three datasets: training (*n* = 60), external validation (ExVal, *n* = 30), and generalizability evaluation (GenEva, *n* = 30) datasets respectively. The patients in the training and ExVal dataset were acquired on the same CT simulator, while those in GenEva were on a different CT simulator. The commercial DLAS software was first localized fine-tuned (LFT) for clinical target volume (CTV) and organs-at-risk (OAR) using the training data, and then validated on ExVal and GenEva respectively. Performance evaluation involved comparing the LFT model with the vendor-provided pretrained model (VPM) against ground truth contours, using metrics like Dice similarity coefficient (DSC), 95th Hausdorff distance (95HD), sensitivity and specificity.

**Results:**

LFT significantly improved CTV delineation accuracy (*p* < 0.05) with LFT outperforming VPM in target volume, DSC, 95HD and specificity. Both models exhibited adequate accuracy for bladder and femoral heads, and LFT demonstrated significant enhancement in segmenting the more complex small intestine. We did not identify performance degradation when LFT and VPM models were applied in the GenEva dataset.

**Conclusions:**

The necessity and potential benefits of LFT DLAS towards institution-specific model adaption is underscored. The commercial DLAS software exhibits superior accuracy once localized fine-tuned, and is highly robust to imaging equipment changes.

## Introduction

Clinical target volume (CTV) and organ-at-risk (OAR) delineation are crucial in successful radiotherapy (RT) treatment planning, with accurate segmentation being vital for delivering safe and effective radiation doses to tumor lesions while minimizing damage to surrounding normal tissues. Conventionally, radiation oncologists manually contour CTV and OAR structures slice by slice, the process of which involves a certain degree of variability. Over the past years, considerable efforts have been made towards developing deep-learning based auto-segmentation (DLAS) models specific for CTV and OAR delineation in radiotherapy. Numerous studies demonstrate promising benefits of DLAS in both accuracy and efficacy over atlas-based methods [1–4]. Also, DLAS’s effectiveness in mitigating dose inconsistencies has been notably observed in a simulation study [5] based on RTOG 0617 [6, 7], a multi-institutional clinical trial, highlighting substantial potential in streamlining and standardizing clinical workflows. Despite proven advantages in DLAS, most advancements predominantly rely on in-house developed DLAS models, and application of DLAS as a routine tool in clinical settings falls far below anticipation. Challenges persist in the clinical adoption of DLAS models, which is highlighted by a survey across 246 institutions where only 26% reported using DLAS in their clinical practice [8].

Generalizability remains a primary challenge for current DL models, where validated models may perform inferiorly in clinical scenarios not represented in their training procedure. For example, deploying top-rated DLAS models from prestigious challenges directly to local institutions resulted in suboptimal performance [9]. Similarly, several studies [10–12] reported notable performance decline when using DL models in external data. Duan et al. [13] evaluated three commercial DLAS products with local cases and observed compromised performance as well.

The mismatching among training and validation datasets, known as *data shift*, contributes significantly to performance deterioration after clinical deployment of DLAS models [14]. Such shifts may result from variations in clinical practices [15], evolution of delineation guidelines [16], or differences in imaging equipment.

To address these issues, upfront model recalibration or adaption is recommended to meet institution-specific standards prior to clinical application [15]. Instead of retraining DLAS models from the ground, a viable and efficient solution is to incrementally retrain or localized fine-tune pretrained models using pooled data to incorporate institution-specific protocols. Balagopal et al. [17] proposed a network model named PSA-Net that segments CTV for postoperative prostate cancer, and observed 5% DSC improvement when adapting to the style of a separate institution.

Fortunately, some commercial vendors offer model retraining services or research tools for users to customize their DLAS models using institution-specific data. However, relevant studies on their clinical implementation is quite limited. Previous studies by Duan et al. [13] and Hobbis et al. [18] investigated fine-tuning a commercial DLAS software (INTContour, CarinaAI) for OAR structures in prostate cancer patients. However, experiences in localized adaptation, particularly for CTV or other tumor sites, remain unexplored.

To this end, this study addresses this notable gap by detailing the process and outcomes of localized fine-tuning and validation of a popular commercial DLAS product for rectal cancer radiotherapy in a clinical setting. The key novelties and contributions of our work are manifold: (1) first study on DLAS model fine-tuning specifically for rectal cancer radiotherapy, (2) specific retrained model has been applied on the basis of a popular DLAS product in mainland China, (3) comprehensive focus on the adaptation and validation of both CTV and OAR structures, and (4) practical insights into model generalizability in the context of changes in imaging equipment- a frequent scenario in clinical settings and we eventually encountered.

## Materials & methods

**Fig. 1:**
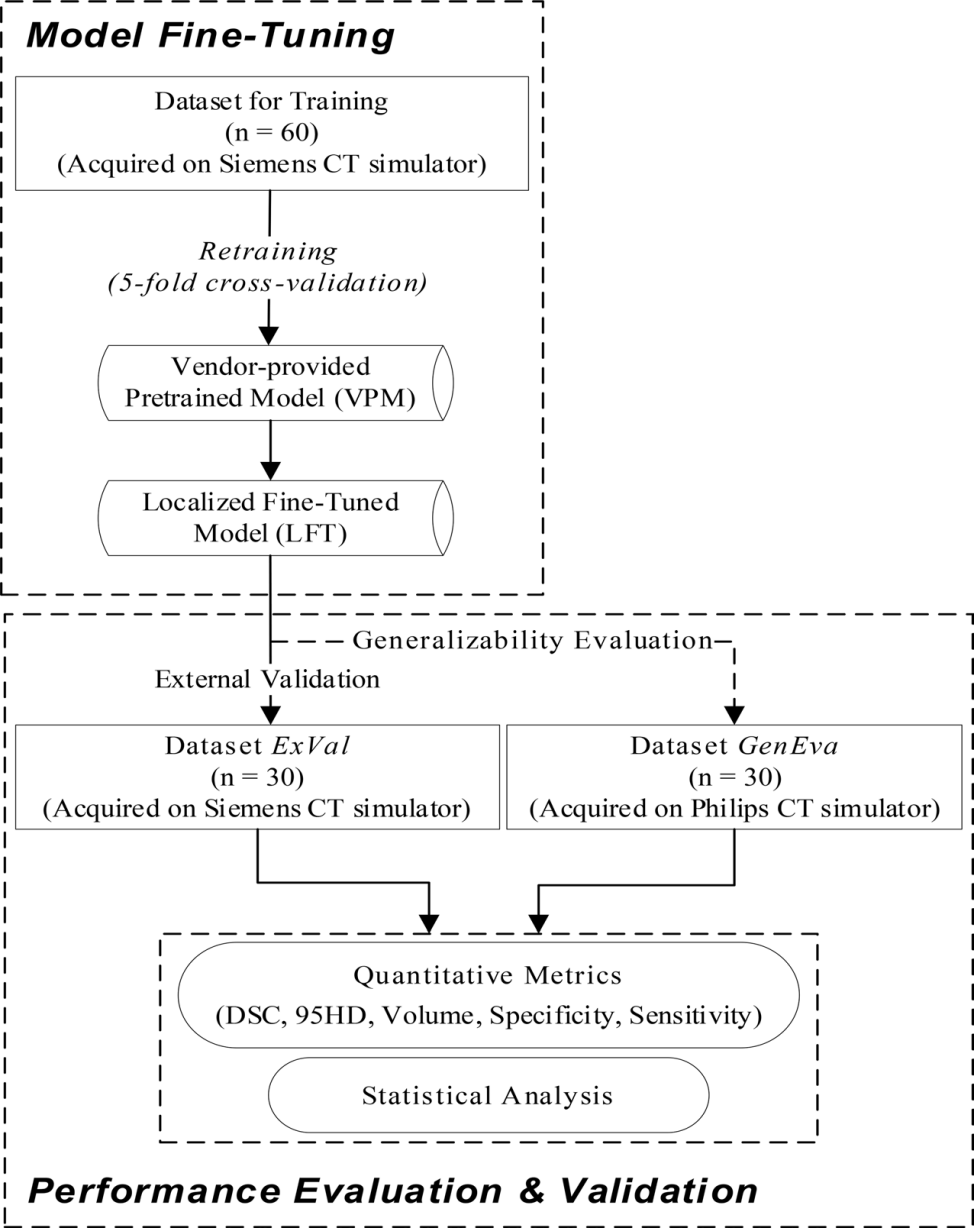
Conceptual design and implementation workflow of this study in model fine-tuning and performance evaluation (external validation and generalizability evaluation)

The conceptual design and overall workflow are shown in Fig. 1. The work is generally composed of two procedures-model fine-tuning, and performance evaluation. The latter includes external validation, evaluating model performance on patients scanned on the same CT simulator as training patients but not utilized during model training. Generalization evaluation refers to assessing the model performance on patients scanned on a different CT simulator and not involved in model training.

### Data collection

#### Patient cohort

This retrospective study was approved by the institutional review board (IRB) of Peking University Cancer Hospital. A total of 120 patients were included in this work, who were diagnosed with Stage II/III mid-low rectal cancer (i.e., gross tumors were located within 10 cm from the anal verge) and received chemoradiation at the institutional radiotherapy department. Over the enrolled cohort, 71 were female and 49 were male, and the ages ranged from 33 to 86 with the median as 65.

The enrolled patients were grouped into three datasets - a training dataset, an external validation dataset denoted as *ExVal* and a generalizability evaluation dataset denoted as *GenEva* as shown in Table 1. The training dataset was composed of 60 patients treated between March 2020 and October 2022. The external validation dataset *ExVal* was composed of 30 patients treated between November 2022 and May 2023. At the end of 2022, a Philips RT-specific CT scanner was commissioned into clinical service at our institution, and 30 patients scanned on this CT-Sim between February 2023 and May 2023 were collected as the dataset *GenEva* to evaluate model generalizability.

**Table 1:** Description of patient data grouping

Dataset name	Cohort volume, female/male	Age (min-max, median)	CT scanner	Image acquisition protocol (kVp, FOV, resolution)
*Training*	60, 21/39	33–83, 62	Siemens Sensation Open CT simulator	120, 65 cm, 1.27 × 1.27 × 5 mm^3^
*External Validation* *(ExVal)*	30, 10/20	37–86, 63	Siemens Sensation Open CT simulator	120, 65 cm, 1.27 × 1.27 × 5 mm^3^
*Generalization Evaluation* *(GenEva)*	30, 9/21	41–83, 66	Philips Big-bore RT simulator	120, 60 cm, 1.17 × 1.17 × 5 mm^3^

#### Image acquisition

In this study, patients were immobilized using a pelvic thermoplastic in a supine position. The training dataset and ExVal were scanned on a Siemens Sensation Open CT simulator, while the GenEva dataset was scanned on a Philips Big-Bore CT simulator. Detailed specifications of the scan parameters are listed. The CT images were imported into the Eclipse Treatment Planning System (Varian Medical System Inc., USA) for physician to delineate target and OAR structures. The contours as well as plans were reviewed by an internal panel before approved for clinical treatment.

In this retrospective study, we retrieved the planning CT images as well as CTV and OAR contours from the treatment planning system in an anonymized approach under the IRB approval. The CTV and OAR contours approved for treatment were used ground truth (GT) reference in model training and performance evaluation. It’s important to emphasize that all the contours used were based on real-world data, and no editing was done to refine them specifically for this study.

### DL model and localized fine-tuning

#### DL kernel network

**Fig. 2:**
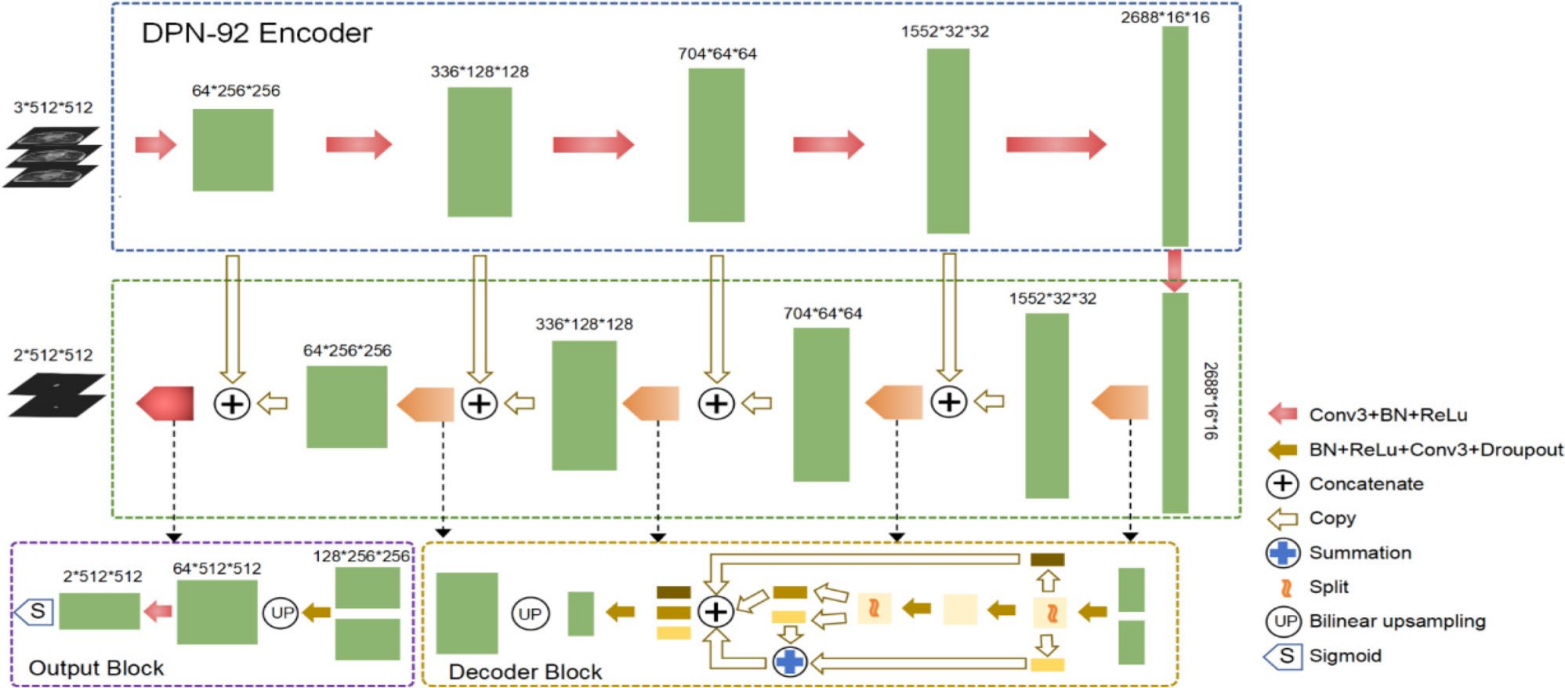
Schematic of the kernel DpnUNet network architecture

The DL model for rectal cancer neoadjuvant radiotherapy herein was adopted from the work by Wu et al. [19, 20] and commercialized as RT-Mind-AI (MedMind Technology Co. Ltd., Beijing, China). The backbone network, referred as DpnUNet, was characterized by integrating dual-path-network (DPN) modules into the UNet structure. The overall architecture of DpnUNet was generally depicted in Fig. 2.

#### Localized model fine-tuning

The model was pretrained using 122 patients’ data from a single institution [19]. We further trained the model with the enrolled training data (60 patients) to adapt to the institutional contouring protocol. The contours of interest were CTV, bladder, femoral heads and small intestine. The class weighted cross-entropy was used to take into account the overall accuracy in both CTV and OARs. Localized model fine-tuning was performed on a single GPU workstation (Nvidia GeForce RTX 2080Ti) using 5-fold cross validation (48 vs. 12). The optimizer was Adam, and the batch size was 4. The initial learning rate was 0.0001, and the value decayed exponentially by a factor of 0.9 over each epoch. The epoch was 60, and the model with the lowest cross-validation loss was selected as the final output.

### Performance evaluation

#### External validation and generalizability evaluation

This study used two datasets (*ExVal* and *GenEva*) with 30 cases in each to evaluate model performance in two aspects. The data in *ExVal* were acquired on the same CT simulator with the training data, and therefore used for external validation. The data in *GenEva* were acquired on a different CT simulator, and herein were used to evaluate model generalization in the context of imaging equipment changes.

#### Quantitative metrics

Two sets of deep learning predicted contours were generated for all 60 testing cases, using a vendor-provided pretrained model (VPM) and a localized fine-tuned model (LFT) respectively. We utilized several valid and widely used metrics to quantify segmentation performance, including the Dice Similarity Coefficient (DSC), the 95th percentile of the Hausdorff Distance (95HD), sensitivity, and specificity, using the clinically approved CTV and OAR contours as GT.

DSC, the most used measure in the field of medical image segmentation, provides an effective assessment of similarity, and is defined as:

1 $$\text{D}\text{S}\text{C}(\text{D},\text{G})=\frac{2\left|\text{D}\cap \text{G}\right|}{\left|\text{D}\right|+\left|\text{G}\right|}$$

where *D* and *G* represent the DLAS-predicted and GT contours respectively, and |*D*∩*G*| represents the intersected volume between *D* and *G.*

The 95HD metric is a routinely used spatial distance-based metric to measures the distance between the DLAS-predicted and GT contours, which is defined as

2 $$95\text{H}\text{D}(\text{D},\text{G})=\text{p}\text{e}\text{r}\text{c}\text{e}\text{n}\text{t}\text{i}\text{l}\text{e}\left(\text{h}\right(\text{D},\text{G})\cup \text{h}(\text{G},\text{D}),95\text{t}\text{h})$$

3 $$\text{h}(\text{D},\text{G})={\text{m}\text{a}\text{x}}_{{\text{d}}_{\text{i}}}\left({\text{m}\text{i}\text{n}}_{{\text{r}}_{\text{j}}}||{d}_{i}- {g}_{j}||\right),{d}_{i}\in \text{D}, {g}_{j}\in \text{G}$$

where ||.|| stands for the Euclidean norm of the points of *d* and *g*.

Sensitivity and specificity are popular metrics for the evaluation of medical image segmentation performance [14, 15], which are defined as

4 $$\text{S}\text{e}\text{n}\text{s}\text{i}\text{t}\text{i}\text{v}\text{i}\text{t}\text{y}=\frac{\text{T}\text{P}}{\text{T}\text{P}+\text{F}\text{N}}$$

5 $$\text{S}\text{p}\text{e}\text{c}\text{i}\text{f}\text{i}\text{c}\text{i}\text{t}\text{y}=\frac{\text{T}\text{N}}{\text{T}\text{N}+\text{F}\text{P}}$$

which TP, FP, TN and FN denote the pixel numbers of true positive, false positive, true negative and false negative respectively for DLAS-predicted CTV and OAR contours, which reflect the number of pixels that are classified correctly or incorrectly with respect to the GT [21].

In addition, the CTV volume was also measured. The DSC, 95HD, sensitivity, specificity, and CTV-volume values of each testing case were calculated in the *3D Slicer* software (version 5.4.0) [16].

#### Statistical analysis

The mean and standard deviation (SD) values were calculated for each metric. Within each testing dataset, the Wilcoxon paired signed-rank test was used to compare the performance between VPM and LFT. The statistical analysis was performed in OriginPro (version 2021a, OriginLab, USA), and the significance level was set at 0.05.

## Results

**Fig. 3:**
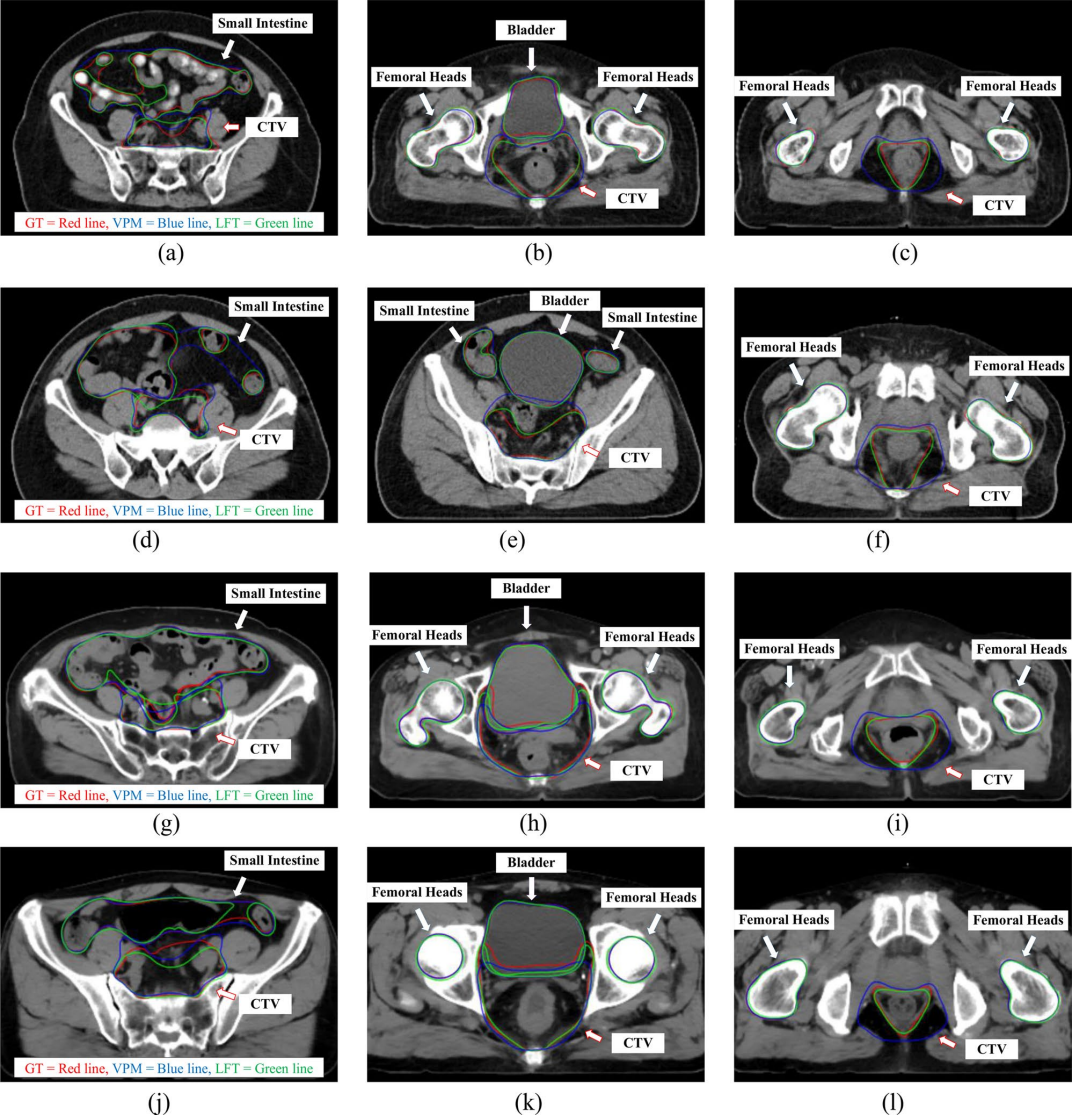
Representative patient cases of CTV and OARs (bladder, femoral head, and small intestine) contours. The upper two rows are cases in *ExVal*, and the lower two rows in *GenEva*. (GT-red line, VPM-blue line, and LFT-green line)

### Visualization of representative cases

Figure 3 shows representative patient cases that are selected from *ExVal* and *GenEva* datasets, acquired on two different imaging devices as shown in Table 1, for subjective visual illustration. Images in *ExVal* (Fig. 3(a-f)) exhibit finer texture patterns, while images in *GenEva* (Fig. 3(g-l)) appear smoother with reduced noise. Also, we can see that CTV and OAR contours predicted either by VPM or LFT models are generally consistent with GT, especially in organs of bladder and femoral heads, where the contours of VPM, LFT and GT are highly overlapped. Regarding the small intestine, although some deviations are shown in Fig. 3(a), (d), and (j), the majority of intestine loops predicted by VPM and LFT closely conform to the ground truth (GT).

### Quantitative assessment of CTV

**Fig. 4:**
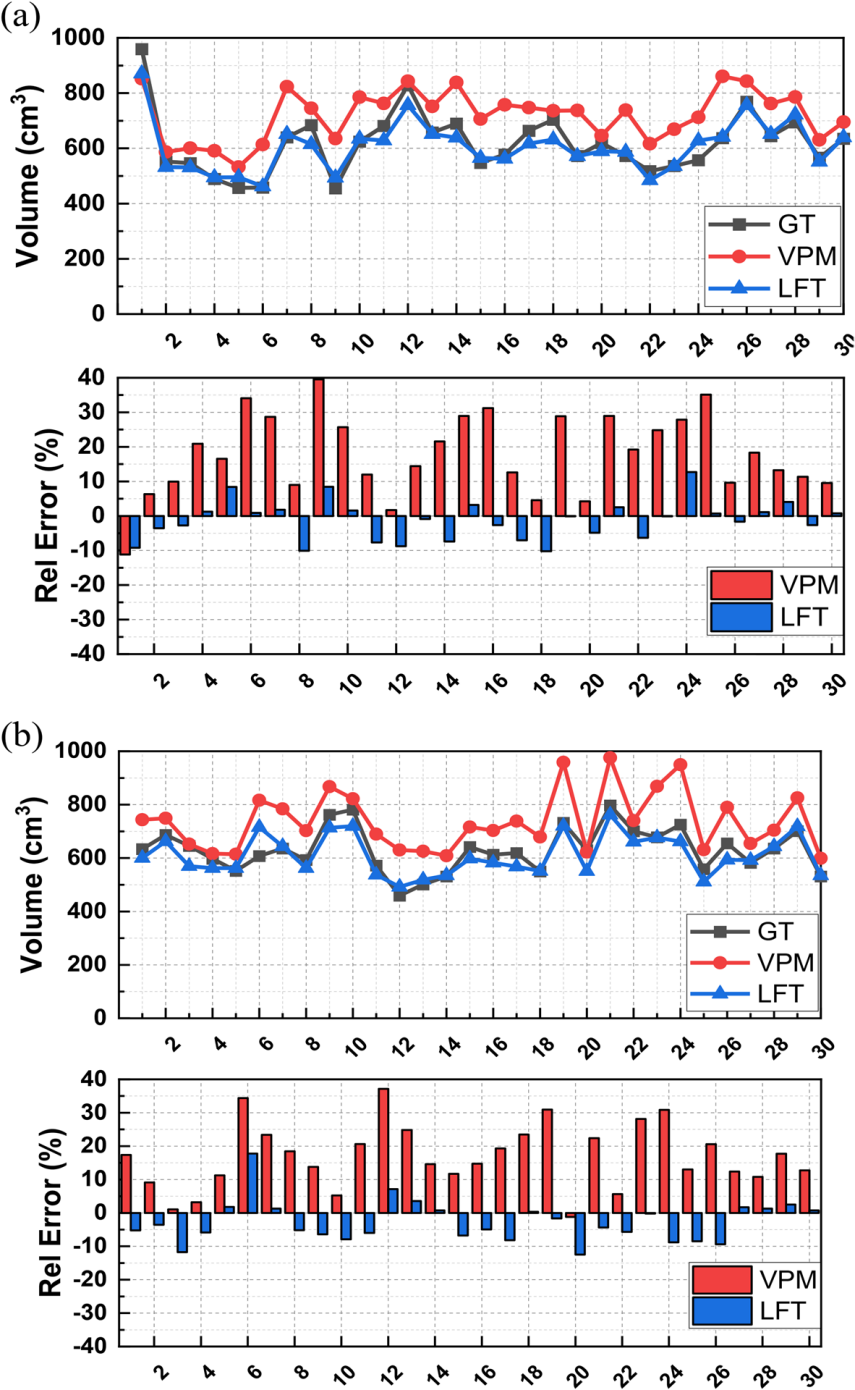
Profile and relative errors of VPM and LFT predicted CTV volumes in comparison with GT over (**a**) *ExVal*, and (**b**) *GenEva* respectively. (GT-black line, VPM-red line/bar, and LFT-blue line/bar)

From Fig. 3, we can also see that, although both VPM and LFT contours are generally consistent with GT, CTV volumes predicated by VPM appear to be larger than either GT or LFT. This *over-contouring* effect is observed in both *ExVal* and *GenEva* datasets and further validated in case-by-case comparison in Fig. 4.

The CTV volumes (mean ± std) predicted by VPM and LFT in *ExVal* are 720.304 ± 90.789 cm^3^ and 606.443 ± 90.677 cm^3^ respectively with the benchmark GT as 617.879 ± 110.506 cm^3^ (p-value < 0.05). The corresponding relative errors in comparison with GT 17.921 ± 11.663% and − 1.281 ± 5.655%. The CTV volumes predicted by VPM and LFT in *GenEva* are 735.997 ± 109.678 cm^3^ and 610.804 ± 74.917 cm^3^ respectively with the benchmark GT as 630.320 ± 82.261 cm^3^ (p-value < 0.05). The corresponding relative errors in comparison with GT 16.923 ± 9.661% and − 2.798 ± 6.228%.

**Fig. 5:**
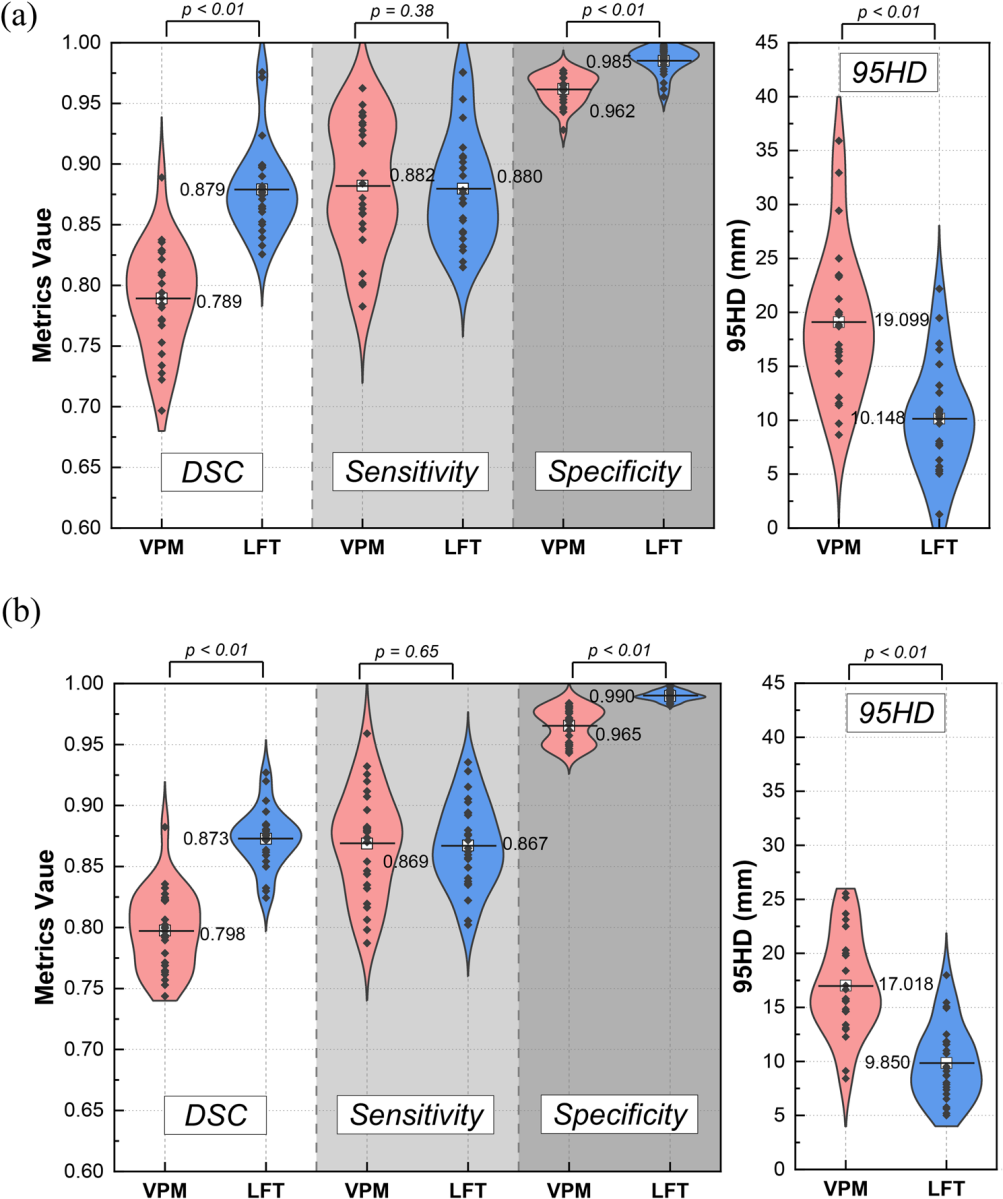
Distribution comparison and statistical analysis of the CTV contours by VPM and LFT models in (**a**) *ExVal* and (**b**) *GenEva* compared with GT in metrics of DSC, sensitivity, specificity and 95HD respectively

Figure 5 shows the distributions of DSC, sensitivity, specificity and 95HD values for DLAS-predicated CTV contours. It indicates that the performance of the LFT model is superior to VPM in metrics of DSC, specificity and 95HD in both *ExVal* and *GenEva* datasets. Specifically, the improvements of DSC mean values are 11.406% in *ExVal* and 9.340% in *GenEva* with statistical significance (p-value < 0.01). The mean specificity values are improved by 2.497% in *ExVal* and 2.591% in *GenEva* (p-value < 0.01), and the mean 95HD values are reduced by 46.866% and 42.120% respectively (p-value < 0.01). On the contrary, the sensitivity distributions between VPM and LFT predicted CTV contours are very close in both *ExVal* and *GenEva* (*p*-value > 0.05).

### Quantitative assessment of OAR

Table 2 summarizes the statistical analysis in metrics of DSC, 95HD, sensitivity and specificity for OAR structures. As for bladder and femoral heads, both VPM and LFT models exhibit sufficient and comparable performance. Despite that some p-values are < 0.05, differences in metrics values between LFT and VPM are negligible, so are those between *ExVal* and *GenEva*. This is consistent with the overlapping contours in Fig. 3, which demonstrates that both VPM and LFT models are adequately accurate in segmenting bladder and femoral heads. As for small intestine, we can see that LFT generally outperformed VPM (p-value < 0.05 in DSC-*GenEva*, 95HD-*GenEva*, sensitivity-*ExVal* and sensitivity-*GenEva*) except in specificity, of which the difference is negligible.

**Table 2:** Summary and statistical analysis of OAR structures (bladder, femoral heads, and small intestine) predicated by VPM and LFT in comparison with GT in metrics of DSC, 95HD, sensitivity and specificity

	DSC		95HD (mm)		Sensitivity		Specificity	
	ExVal	GenEva	ExVal	GenEva	ExVal	GenEva	ExVal	GenEva
Bladder								
* VPM*	0.967 ± 0.013	0.964 ± 0.015	2.837 ± 1.465	3.311 ± 1.723	0.972 ± 0.021	0.987 ± 0.017	0.996 ± 0.010	0.997 ± 0.08
* LFT*	0.961 ± 0.011	0.961 ± 0.014	3.327 ± 1.065	3.582 ± 1.881	0.965 ± 0.020	0.957 ± 0.028	0.992 ± 0.013	0.992 ± 0.017
* p-value*	< 0.01	< 0.01	0.32	0.51	0.02	< 0.01	0.03	< 0.01
Femoral heads								
* VPM*	0.956 ± 0.013	0.972 ± 0.011	2.193 ± 0.573	1.922 ± 0.736	0.941 ± 0.019	0.972 ± 0.013	0.999 ± 0.001	0.999 ± 0.001
* LFT*	0.949 ± 0.008	0.970 ± 0.013	2.478 ± 0.532	1.978 ± 0.515	0.961 ± 0.012	0.976 ± 0.012	0.998 ± 0.001	0.998 ± 0.001
* p-value*	0.01	0.14	0.03	0.62	< 0.01	0.03	< 0.01	< 0.01
Small intestine								
* VPM*	0.838 ± 0.072	0.806 ± 0.068	8.312 ± 2.821	11.605 ± 3.153	0.779 ± 0.121	0.723 ± 0.097	0.992 ± 0.005	0.993 ± 0.005
* LFT*	0.853 ± 0.051	0.834 ± 0.084	8.201 ± 3.229	10.888 ± 3.430	0.835 ± 0.096	0.808 ± 0.121	0.980 ± 0.017	0.985 ± 0.012
* p-value*	0.13	< 0.01	0.91	0.03	< 0.01	< 0.01	< 0.01	< 0.01

## Discussion

In this study, we meticulously detailed our process and outcomes from localized fine-tuning and validation of a popular commercial DLAS product, RT-Mind-AI, specifically targeting rectal cancer radiotherapy. This work marks a significant stride in not only addressing the imperative need in enhancing DLAS model performance in real-word clinical settings but also the generalizability of RT-Mind-AI in the context of imaging equipment changes.

In the process of localized model fine-tuning, we used real-world patient data that had been approved for clinical treatment with minimal data preprocessing. The training cohort was composed of 60 patients, much smaller than pertinent studies on in-house DLAS model development (135 patients in Wu Y et al. [19], 218 patients in Men K et al. [22], 136 patients in Song Y et al. [23], 100 patients in Larsson R et al. [24]) and on incrementally training a commercial DLAS model in [13] (with 100 patients) as well. The use of a small volume of real-world patient data has underscored the practicality and efficiency of on-site data preparation, which facilitates the localized fine-tuning process without extensive data collection and labor-intensive data preprocessing.

The CTV evaluation results demonstrate that the fine-tuned RT-Mind-AI exhibited comparable performance with in-house models (mean DSC = 0.879 in *Exval* /0.874 in *GenEva*, 0.90 in Wu Y et al. [19], 0.877 in Men K et al. [22], 0.88 in Song Y et al. [23], 0.90 in Larsson R et al. [24]). Notably, the LFT model showed superior performance over the VPM model, especially in the accuracy of CTV volume estimation and the reduction of over-contouring tendencies. The substantial improvements in metrics such as DSC and 95HD highlight the effectiveness of adapting models to institution-specific clinical standards.

For bladder and femoral heads, the segmentation performance of both VPM and LFT was adequately accurate, as indicated by the high metrics values. This finding indicates that the vendor-provided model has been well trained and these model components may not require further retraining, potentially easing the implementation process in clinical settings. This indication is consistent with the study by Hobbis D et al. [18]. This is mainly due to the distinct anatomical characteristics of bladder and femoral heads and lower inter-observer variability among physicians. Accurate segmentation of the small intestine poses a significantly greater challenge than that of the bladder and femoral heads. Although the LFT method has shown significant improvements in DSC, 95HD and sensitivity metrics (p-value < 0.05), these metrics still fall short of the benchmarks established in bladder and femoral heads segmentation. This discrepancy is primarily due to two factors. Firstly, the small intestine’s anatomy is inherently complex, exhibiting considerable variability in positioning and filling of the intestines, which makes distinguishing between intestinal loops and adjacent tissues particularly difficult. Secondly, the variation in contouring the small intestine among different observers is significant [13, 18], making it challenging to achieve a strictly consistent standard. This also highlights the importance of enhancing DLAS accuracy for complex anatomical structures.

Equipment changes are significant events that may take place occasionally in clinical practice, and one of the significant aspects of this study is addressing the impact of different CT simulators on model performance. The robustness of the commercial DLAS product, even with changes in imaging equipment, is a promising finding for institutions undergoing technological upgrades or those using multiple imaging systems. This robustness is crucial for the widespread adoption of DLAS technologies, ensuring consistent performance across various clinical environments.

Despite these promising results, we acknowledge several limitations in this work. First, this study is based on a specific commercial DLAS product (RT-Mind-AI) and two CT simulators. As for other DLAS products or simulators, some findings may not be solidly valid. The generalizability of these results to other DLAS products or imaging equipment needs further investigation. Second, the cohort size for model fine-tuning was empirical based on our previous work in Geng J et al. [25]. Maybe a smaller cohort would be enough, and future studies might explore the optimal cohort size for effective and efficient model fine-tuning. Third, this study is focused on rectal cancer radiotherapy. Next-step efforts will be directed to DLAS model fine-tuning for other tumor sites to facilitate clinical application as well as further validation.

## Conclusion

We detailed the process and outcomes from localized fine-tuning and validation of a popular commercial DLAS product (RT-Mind-AI) specifically for rectal cancer radiotherapy in real-world clinical settings. The comprehensive validation explicitly underscores the necessity and potential benefits in institution-specific DLAS model adaption and continues model updating, which indicates that localized model fine-tuning for various clinical settings is crucial in realizing the full potential of DLAS in enhancing the precision and effectiveness of radiotherapy treatments. This work also demonstrate that the RT-Mind-AI software is highly robust to imaging equipment changes, and exhibits superior accuracy once localized fine-tuned.

## Data Availability

The data that support this study are not openly available due to ethical and privacy concerns but are available from the corresponding author upon reasonable request.

## Abbreviations

95HD: 95th percentile Hausdorff distance
CNN: Convolutional neural network
CT: Computed tomography
CTV: Clinical target volume
DL: Deep-learning
DLAS: Deep learning auto-segmentation
Dpn: Dual-path network
DSC: Dice similarity coefficient
GT: Ground truth
GTV: Gross tumor volume
LFT: Localized fine-tuned
OAR: Organ-at-risk
ROI: Region of interest
RT: Radiotherapy
SD: Standard deviation
VPM: Vendor-provided pretrained

## References

[1] Chen W, Li Y, Dyer BA, Feng X, Rao S, Benedict SH (2020). Deep learning vs. atlas-based models for fast auto-segmentation of the masticatory muscles on head and neck CT images. Radiat Oncol.

[2] Seo H, Badiei Khuzani M, Vasudevan V, Huang C, Ren H, Xiao R (2020). Machine learning techniques for biomedical image segmentation: an overview of technical aspects and introduction to state-of-art applications. Med Phys.

[3] Vrtovec T, Močnik D, Strojan P, Pernuš F, Ibragimov B (2020). Auto-segmentation of organs at risk for head and neck radiotherapy planning: from atlas-based to deep learning methods. Med Phys.

[4] Bandyk MG, Gopireddy DR, Lall C, Balaji KC, Dolz J (2021). MRI and CT bladder segmentation from classical to deep learning based approaches: current limitations and lessons. Comput Biol Med.

[5] Thor M, Apte A, Haq R, Iyer A, LoCastro E, Deasy JO (2021). Using auto-segmentation to reduce contouring and dose inconsistency in clinical trials: the simulated impact on RTOG 0617. Int J Radiat Oncol Biol Phys.

[6] Bradley JD, Paulus R, Komaki R, Masters G, Blumenschein G, Schild S (2015). Standard-dose versus high-dose conformal radiotherapy with concurrent and consolidation carboplatin plus paclitaxel with or without cetuximab for patients with stage IIIA or IIIB non-small-cell lung cancer (RTOG 0617): a randomised, two-by-two factorial phase 3 study. Lancet Oncol.

[7] Bradley JD, Hu C, Komaki RR, Masters GA, Blumenschein GR, Schild SE (2020). Long-term results of NRG Oncology RTOG 0617: standard- Versus High-Dose Chemoradiotherapy with or without Cetuximab for Unresectable Stage III Non-small-cell Lung Cancer. J Clin Oncol off J Am Soc Clin Oncol.

[8] Machine learning applications in radiation oncology (2020). Current use and needs to support clinical implementation. Phys Imaging Radiat Oncol.

[9] Feng X, Bernard ME, Hunter T, Chen Q (2020). Improving accuracy and robustness of deep convolutional neural network based thoracic OAR segmentation. Phys Med Biol.

[10] Wang X, Liang G, Zhang Y, Blanton H, Bessinger Z, Jacobs N (2020). Inconsistent performance of Deep Learning models on Mammogram classification. J Am Coll Radiol JACR.

[11] Wang B, Dohopolski M, Bai T, Wu J, Hannan R, Desai N et al. Performance deterioration of Deep Learning models after Clinical Deployment: a case study with auto-segmentation for definitive prostate Cancer Radiotherapy 2023. 10.48550/arXiv.2210.05673.

[12] Pan I, Agarwal S, Merck D (2019). Generalizable inter-institutional classification of abnormal chest radiographs using efficient convolutional neural networks. J Digit Imaging.

[13] Duan J, Vargas CE, Yu NY, Laughlin BS, Toesca DS, Keole S (2023). Incremental retraining, clinical implementation, and acceptance rate of deep learning auto-segmentation for male pelvis in a multiuser environment. Med Phys.

[14] Finlayson SG, Subbaswamy A, Singh K, Bowers J, Kupke A, Zittrain J (2021). The clinician and dataset shift in Artificial Intelligence. N Engl J Med.

[15] Roper J, Lin M-H, Rong Y (2023). Extensive upfront validation and testing are needed prior to the clinical implementation of AI-based auto-segmentation tools. J Appl Clin Med Phys.

[16] Brouwer CL, Steenbakkers RJHM, Gort E, Kamphuis ME, van der Laan HP, Van’t Veld AA (2014). Differences in delineation guidelines for head and neck cancer result in inconsistent reported dose and corresponding NTCP. Radiother Oncol J Eur Soc Ther Radiol Oncol.

[17] Balagopal A, Morgan H, Dohopolski M, Timmerman R, Shan J, Heitjan DF (2021). PSA-Net: deep learning–based physician style–aware segmentation network for postoperative prostate cancer clinical target volumes. Artif Intell Med.

[18] Hobbis D, Yu NY, Mund KW, Duan J, Rwigema J-CM, Wong WW (2023). First Report on Physician Assessment and Clinical Acceptability of Custom-Retrained Artificial Intelligence models for clinical target volume and organs-at-risk auto-delineation for Postprostatectomy patients. Pract Radiat Oncol.

[19] Wu Y, Kang K, Han C, Wang S, Chen Q, Chen Y (2022). A blind randomized validated convolutional neural network for auto-segmentation of clinical target volume in rectal cancer patients receiving neoadjuvant radiotherapy. Cancer Med.

[20] Liu Z, Liu X, Guan H, Zhen H, Sun Y, Chen Q (2020). Development and validation of a deep learning algorithm for auto-delineation of clinical target volume and organs at risk in cervical cancer radiotherapy. Radiother Oncol.

[21] Dominik M, Iñaki SR, Frank K (2022). Towards a guideline for evaluation metrics in medical image segmentation. BMC Res Notes.

[22] Men K, Dai J, Li Y (2017). Automatic segmentation of the clinical target volume and organs at risk in the planning CT for rectal cancer using deep dilated convolutional neural networks. Med Phys.

[23] Song Y, Hu J, Wu Q, Xu F, Nie S, Zhao Y (2020). Automatic delineation of the clinical target volume and organs at risk by deep learning for rectal cancer postoperative radiotherapy. Radiother Oncol.

[24] Larsson R, Xiong J-F, Song Y, Ling-Fu, Chen Y-Z, Xiaowei X, et al. Automatic delineation of the clinical target volume in rectal Cancer for Radiation Therapy using three-dimensional fully convolutional neural networks. 2018 40th Annu. Int Conf IEEE Eng Med Biol Soc EMBC. 2018;5898–901. 10.1109/EMBC.2018.8513506. 10.1109/EMBC.2018.8513506 30441678

[25] Geng J, Zhang S, Wang R, Bai L, Chen Q, Wang S (2024). Deep-learning based triple-stage framework for MRI-CT cross-modality gross tumor volume (GTV) segmentation for rectal cancer neoadjuvant radiotherapy. Biomed Signal Process Control.

